# Adipose tissue-derived exosomes alleviate particulate matter-induced inflammatory response and skin barrier damage in atopic dermatitis-like triple-cell model

**DOI:** 10.1371/journal.pone.0292050

**Published:** 2024-01-19

**Authors:** Yoon Jin Roh, Yong Hee Choi, Sun Hye Shin, Mi-Kyung Lee, Yu Jin Won, Jun Ho Lee, Byong Seung Cho, Kui Young Park, Seong Jun Seo

**Affiliations:** 1 Department of Dermatology, Chung-Ang University College of Medicine, Seoul, Korea; 2 Department of Laboratory Medicine, Chung-Ang University College of Medicine, Seoul, Korea; 3 ExoCoBio Exosome Institute (EEI), ExoCoBio Inc., Seoul, Korea; Sungkyunkwan University and Samsung Advanced Institute of Health Science and Technology (SAIHST), REPUBLIC OF KOREA

## Abstract

Recently, particulate matter (PM) has been shown to exacerbate atopic dermatitis (AD) by inducing an inflammatory response. Meanwhile, several studies revealed that exosomes derived from adipose tissue-derived mesenchymal stem cells promote wound healing and alleviate inflammation via their regenerative and immunomodulatory capacities. Our study aimed to investigate the effects of human adipose tissue-derived mesenchymal stem cell-derived (ASC)-exosomes in PM-induced AD. An AD-like triple-cell model was established by treating human keratinocytes, dermal fibroblasts, and mast cells with polyinosinic:polycytidylic acid (Poly I:C) and interleukin 1 alpha (IL-1α). The effects of PM and ASC-exosomes on the expression of pro-inflammatory cytokines and skin barrier proteins were examined using quantitative real-time polymerase chain reaction, western blotting, and immunofluorescence. PM increased pro-inflammatory cytokines (IL-6, IL-1β, and IL-1α) and decreased the anti-inflammatory cytokine IL-10, while the mRNA expression of skin barrier proteins (loricrin and filaggrin) decreased. However, when the cells were treated with ASC-exosomes, the PM-induced effects on pro-inflammatory cytokines and skin barrier proteins were reversed. Our results confirmed that PM-induced inflammation and skin barrier damage were alleviated by ASC-exosomes in our AD-like triple-cell model. These data suggest that ASC-exosomes can serve as a therapeutic agent for PM-exacerbated AD.

## Introduction

Atopic dermatitis (AD) is a relatively common inflammatory skin disease [1, 2] and is associated with skin barrier dysfunction and Th2-mediated dysregulated immune responses [3–5]. Although the etiology of AD is not clearly elucidated, air pollution is believed to be a factor in the recent increase in AD incidence [6, 7]. Particulate matter (PM) is a key constituent of air pollution and a major concern worldwide. The contribution of PM exposure to health problems [8, 9], such as cardiovascular, nervous, immune, and respiratory diseases, is increasingly becoming an area of interest [5, 9]. For example, the effects of PM (e.g., fine dust) on skin diseases have been studied more often in recent years [10–12]. Our laboratory’s previous research has shown that PM is involved in skin barrier defects and abnormal immune responses [12–16], and we advocated for the development of therapeutic agents for skin barrier repair and skin inflammation reduction in response to PM exposure [12, 15, 17].

Mesenchymal stem cells (MSCs), which can be harvested from various adult tissues [17, 18], are promising cell types for developing treatments for multiple diseases due to their pluripotency and self-renewal abilities [19]. Adipose tissue-derived MSCs (ASCs) are relatively easy to obtain and isolate and have been used in cell-based therapies [17, 20, 21]. However, ASCs also have limitations, such as low engraftment efficiency, a relatively short half-life, and difficulties implementing quality control procedures [18, 20–22].

Exosomes are 30–200 nm bioactive vesicles released by the fusion of multi-vesicular bodies (late endosomes) with the cell membrane [18, 20]. Depending on the source cell type, exosomes contain various proteins, nucleic acids, and lipids [18]. Exosomes participate in cell-to-cell communication by transferring those cargoes to recipient cells [18, 23]. Thus, exosomes have been widely studied as potential biomarkers for clinical diagnoses and drug delivery systems [23–25]. Furthermore, exosomes derived from stem cells have been shown to exhibit biological effects of stem cells, and therefore the use of stem cell-derived exosomes has been widely studied in the field of dermatology [26, 27]. Recently, ASC-exosomes have been suggested as a potential therapeutic option for AD [18, 28]. A preclinical study by Cho et al. showed that intravenous or subcutaneous administration of human ASC-derived exosomes significantly reduced AD-like symptoms and lowered the expression of Th2 cytokines, including interleukin (IL)-4 and IL-31 in an AD murine model [28]. In addition, Shin et al. reported that ASC-derived exosomes positively modulate barrier functions in an oxazolone-induced AD murine model by increasing the production of ceramides and dihydroceramides and the formation of lamellar bodies [18]. However, the effects of ASC-derived exosomes on AD remain poorly understood. In this study, the effectiveness and mechanisms of ASC-derived exosomes on AD exacerbation by air pollutant PM were investigated using an AD-like cell model [12, 29–31].

## Materials and methods

### Materials

The standard reference materials (SRM) 1649b were purchased from the National Institute of Standards and Technology (NIST, Gaithersburg, MD, USA). PM particles at concentrations of 25 μg/cm^2^ were prepared in serum-free medium.

Interleukin-1 alpha (IL-1α) and polyinosinic:polycytidylic acid sodium salt (Poly I:C), used to produce the AD-like cell model, were obtained from PeproTech (Cranbury, NJ, USA) and Sigma-Aldrich (St Louis, MO, USA). For western blotting, an anti-IL-4 antibody was obtained from Thermo Fisher Scientific (Waltham, MA, USA). An anti-IL-6 antibody was purchased from Cell Signaling (Danvers, MA, USA). An anti-IL-1β antibody was purchased from MyBioSource (San Diego, CA, USA). An anti-GAPDH antibody was obtained from Santa Cruz (Delaware Avenue, CA, USA). An anti-FLG antibody was purchased from Life Span Biosciences (Seattle, WA, USA). Anti-rabbit IgG, anti-mouse IgG, and HRP-linked antibodies were purchased from Cell Signaling Technology. The human immortalized keratinocytes (HaCaT) and human dermal fibroblast (HDF) cell lines were obtained from the Korean Cell Line Bank (Seoul, Korea). The human mast cell line HMC-1 was obtained from Merck Millipore (Temecula, CA, USA).

### Cell culture

HaCaT and HDF cells were cultured in Dulbecco’s modified Eagle’s medium (DMEM) supplemented with 10% heat-inactivated fetal bovine serum (FBS) at 37°C in a humidified 5% CO_2_ atmosphere. In the same incubator, HMC-1 cells were cultured in Iscove’s Modified Dulbecco’s Medium (IMDM, all from Gibco, Grand Island, NY, USA) supplemented with 10% FBS, 2 mM L-glutamine, 1.2 mM α-thioglycerol (all from Sigma) and streptomycin (50 μg/mL). In the triple-cell culture model, HaCaT, HDF, and HMC-1 cells were cultured at a ratio of 1:3:3. The cell medium was changed every 2~3 days. The triple-cell culture medium included DMEM:IMDM at a ratio of 3:1. When the cultures reached confluence, they were treated with Trypsin-EDTA Solution (1X) (Gibco) for 3–5 minutes. In addition, the cells were starved for at least six hours before use.

### ASC-conditioned media

After obtaining written informed consent, human adipose tissue from a healthy donor was obtained from ExoCoBio Inc. (Seoul, Korea) with the approval of the Institutional Review Board of CHA University Medical Center, Korea (IRB No. CHAMC 2019-05-040-018) and evaluated according to the Korean Ministry of Food and Drug Safety (MFDS) guidelines. Characterization of ASCs was performed using previously described methods. In brief, the surface markers for ASCs were determined by flow cytometry, and trilineage differentiation (osteogenic, adipogenic, and chondrogenic differentiation) potencies of ASCs were determined [21]. After isolating ASCs from the adipose tissue, they were subcultured with Minimum essential medium (MEM)-α containing 10% FBS at a density of 3 × 10^3^ cells/cm^2^ at 37°C under 5% CO_2_ and 95% air. After, the cells were disassociated using Trypsin-EDTA solution 1X (Gibco) and then washed with Dulbecco’s phosphate-buffered saline (DPBS; Gibco). Cell stocks of passage four were stored in liquid nitrogen. After thawing, the cell preparation was subcultured until passage seven to generate ASC-conditioned media (CM). ASCs at passage seven were seeded at a density of 6 × 10^3^ cells/cm^2^ and maintained with MEM-α at 37°C under 5% CO_2_ and 95% air for three days up until 80–90% confluence. Finally, the cells were washed DPBS and maintained in serum free- and phenol red-free MEM-α. CM was collected after 24 hours of incubation. MEM-α medium was used for adipocyte proliferation and ASC conditioned media production.

### ASC-exosomes isolation

ASC-exosomes were isolated from the ASC CM using tangential flow filtration (TFF)-based ExoSCRT technology as previously described [18, 28]. During the isolation process, the excipient EDB1 generated by ExoCoBio, Inc., was added for the uniformity of exosome particle size. The CM was filtered by a polyethersulfone (PES) membrane filter (0.22 μm, Merck Millipore, Billerica, MA, USA) to remove contaminating particles, such as cell debris, microvesicles, and apoptotic bodies. The CM was concentrated by a tangential flow filtration membrane cartridge (GE Healthcare, Chicago, IL, USA) with a molecular weight cut-off of 500 kDa, and then buffer exchange to DPBS was carried out by diafiltration. The amount of protein in the isolated ASC-exosomes was approximately 0.5% of the amount in the CM. Isolated ASC-exosomes were stored at -80°C. The frozen ASC-exosomes underwent a single free-thaw cycle before use, and the characterization and profile analysis were performed according to the Minimal Information for Studies of Extracellular Vesicles 2018 [32].

### Reagent and exosome treatment of the cells

The triple cells were seeded at 1.5 × 10^5^ cells per well (2-mL cell suspension of HaCaT, HDF, and HMC-1 cells 1:3:3) in a six-well plate and incubated overnight. The triple-cell culture was pretreated with Poly I:C (10 μg/mL) and IL-1α (10 ng/mL) to induce an AD-like phenotype. After 24 hours, the cell cultures were treated with ASC-exosomes and PM at a concentration of 25 μg/cm^2^, according to previous studies [7, 8, 12]. The plates were incubated at 37°C for an additional 24 hours. Then, the medium was removed, the cells were washed with DPBS, and the cells were treated again with ASC-exosomes.

### Cellular uptake assay

Fluorescence microscopy was used to measure the cellular uptake of the ASC-exosomes. We seeded the HaCaT, HDF, and HMC-1 cells into a 35Ø dish and allowed the cells to attach. The medium was replaced with the DMEM:IMDM serum-free media. The ASC-exosomes were labeled with the fluorescent membrane dye PKH67 (Sigma-Aldrich) and washed in PBS. PKH67-labeled exosomes were added to each 35 Ø dishes at different times (6, 24, 48 h) and incubated with the cells. After the removal of the cell medium, the cells were washed with DPBS. The cells were then fixed with a 10% formalin solution (Biosesang, Seoul, Korea) and counterstained with CellMask^™^ plasma membrane stain (Thermo Fisher Scientific) and Hoechst 33258 nucleic acid stain (Sigma-Aldrich). Finally, we imaged the cells under a fluorescence microscope to visualize the intracellular uptake of the exosomes, with red fluorescence indicating the cell membrane and green fluorescence indicating the ASC-exosomes.

### Quantitative real-time PCR (qRT-PCR)

Total RNA was isolated from the triple cells using the TRIzol^®^ reagent (Invitrogen, Waltham, MA, USA) and quantified following the manufacturer’s instructions. cDNA synthesis for quantitative real-time PCR (qPT-PCR) was conducted using the RevertAid First Strand cDNA synthesis kit (Thermo Fisher Scientific) with 1 μg of total RNA. qRT-PCR was performed using the PowerUp SYBR Green Master Mix (Applied Biosystems, Waltham, MA, USA) and real-time PCR detection system (Applied Biosystems). The experiments were repeated three times, and the results were normalized to GAPDH. Relative expression was calculated using the comparative 2^-ΔΔCt^ method according to the manufacturer’s instructions.

### Western blot analysis

The triple cells were lysed in radioimmunoprecipitation assay (RIPA; Thermo Fisher Scientific) buffer containing a protease and phosphatase inhibitor cocktail (Invitrogen, Waltham, MA, USA). For FLG only, an NP-40 cell lysis buffer (Invitrogen) containing PMSF and a protease inhibitor cocktail was used. The lysates were incubated on ice for 20 minutes and centrifuged at 13,200 rpm for 20 minutes. The protein concentrations were measured using a bicinchoninic acid (BCA, Thermo Fisher Scientific) assay, and the absorbance was measured at 562 nm using a microplate reader. Protein (20 μg) was loaded onto 15% sodium dodecyl sulfate-polyacrylamide gel electrophoresis (SDS-PAGE) and then transferred to a nitrocellulose (NC) membrane. The membrane was then blocked for one hour in Tris-buffered saline containing Tween-20 (TBST) with 5% skim milk. Subsequently, the membrane was incubated with primary antibodies (1:1000) overnight at 4°C and HRP-conjugated secondary antibodies (1:5000) for one hour at room temperature. Finally, the proteins were visualized using Amersham ECL (Cytiva, Thermo Fisher Scientific), and protein bands were visualized using the ChemiDoc XRS system with images (Bio-Rad, Hercules, CA, USA). The protein band intensities were quantitated using ImageJ software (ImageJ, National Institutes of Health, Bethesda, MD, USA). Experiments were repeated three times to confirm the results.

### Immunofluorescence

Cells were fixed using 4% paraformaldehyde in cold DPBS and permeabilized using 10.5% Triton X-100 in DPBS for three minutes. A blocking solution with 5% bovine serum albumin (BSA) was then added. The cells were incubated with IL-6, FLG, IL-4, and IL-1β primary antibodies overnight, followed by incubation with a FITC-labeled secondary goat anti-Rabbit IgG (H&L) antibody (Abcam) for one hour. Nuclei were stained with 4′,6-Diamidino-2-phenylindole, dihydrochloride (DAPI) included in the mounting medium (Abcam). After, all cells were visualized using a Leica SP8 confocal microscopy (Philadelphia. PA, USA).

### Statistical analysis

To compare the resulting exosome effects in the treatment and control samples, we used a one-way analysis of variance and Tukey’s post hoc multiple comparison. All data were statistically analyzed using GraphPad Prism 7 (GraphPad by Dotmatics; San Diego, CA, USA).

## Results

### Analysis and characterization of ASC-exosomes

To analyze the concentration and size of ASC-exosomes, we first measured the particle concentration and size distribution using nanoparticle tracking analysis (NTA) with the Nanosight NTA NS300 instrument (Malvern Instruments, United Kingdom). The results indicated that the mode size of ASC-exosomes is around 130 nm (S1 Fig). Furthermore, we performed a cryo-transmission electron microscopy analysis (Cryo-TEM, Thermo Fisher Scientific) to identify the structure and morphology of ASC-exosomes. This confirmed the presence of clearly discernible lipid bilayer structures as expected (S2 Fig). To further characterize the ASC-exosomes, flow cytometry analysis was performed to test the presence of markers known to be enriched in exosomes, including CD9, CD63, and CD81. The ASC-exosomes were shown to express CD9, CD63, and CD81 (S3 Fig).

### The cellular uptake levels of ASC-exosomes in an AD-like triple-cell model

**Fig 1:**
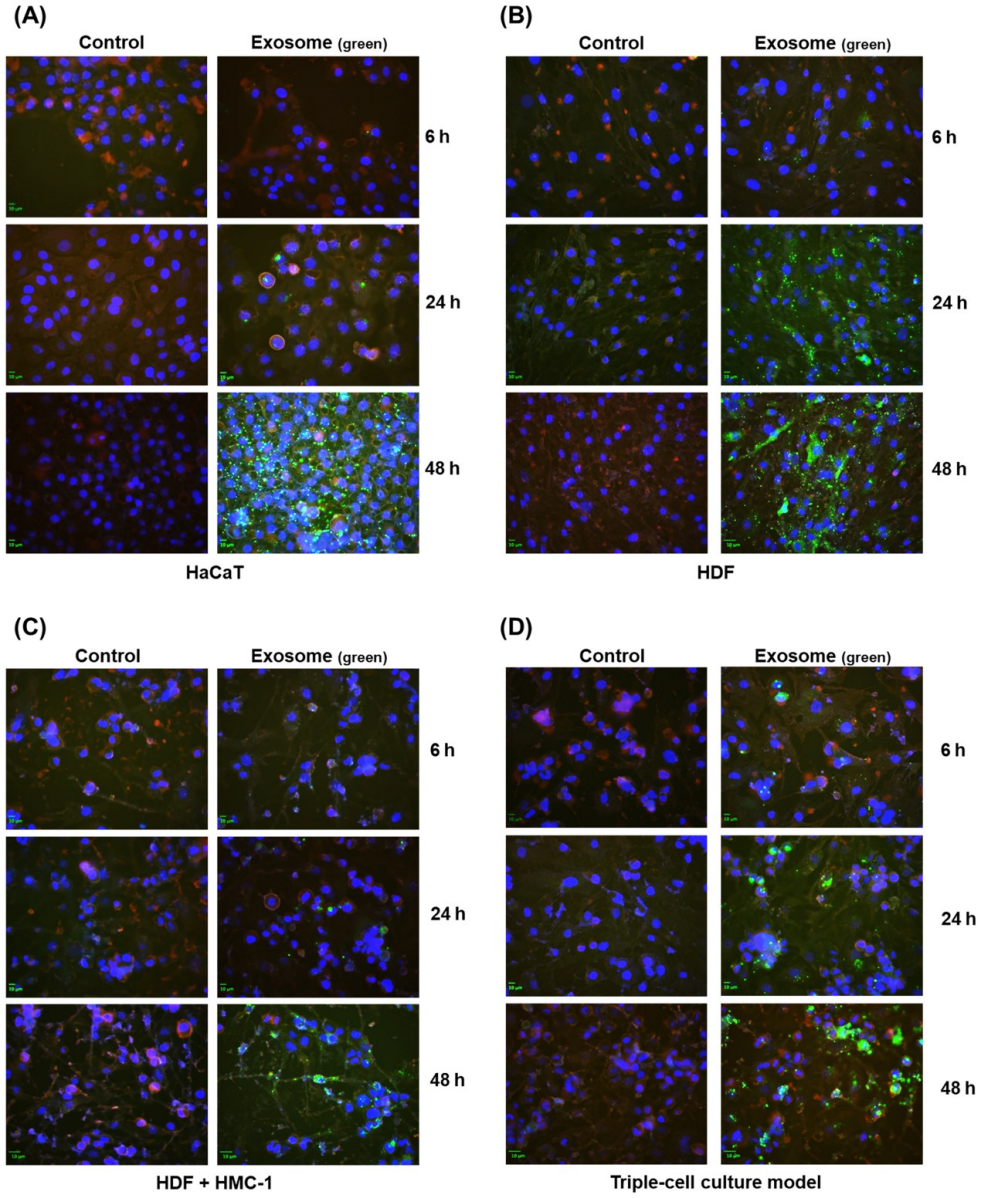
Cellular uptake of adipose stem cell-derived exosomes. Fluorescence microscopy images of the intracellular uptake of ASC-exosomes (green) in the cells (plasma membrane stained red) after treatment for 6, 24, and 48 hours. Nuclei are labeled with Hoechst 33258 (blue). (A) HaCaT cells (Human Keratinocyte); (B) HDF cells (Human Primary Dermal Fibroblasts); (C) HDF + HMC-1 (Human mast cell); (D) Triple-cell culture model.

An AD-like triple-cell model with three different cell types, including HaCaT, HDF, and HMC-1 cells, was used to investigate the cell-to-cell interactions associated with AD [12]. We compared the intracellular uptake of PKH67-labeled ASC-exosomes in HaCaT cells (Fig 1A), HDF cells (Fig 1B), an HDF and HMC-1 cell co-culture (Fig 1C), and the triple-cell culture system (Fig 1D) at 6-, 24-, and 48-hours using fluorescence microscopy. The highest intracellular uptake of green fluorescence occurred at 48 hours rather than 24 hours in all cells (Fig 1). Therefore, the treatment time of the exosomes was selected as 24 and 48 hours.

### ASC-exosomes suppress the mRNA expression of proinflammatory cytokines and skin barrier genes in an AD-like triple-cell model

**Fig 2:**
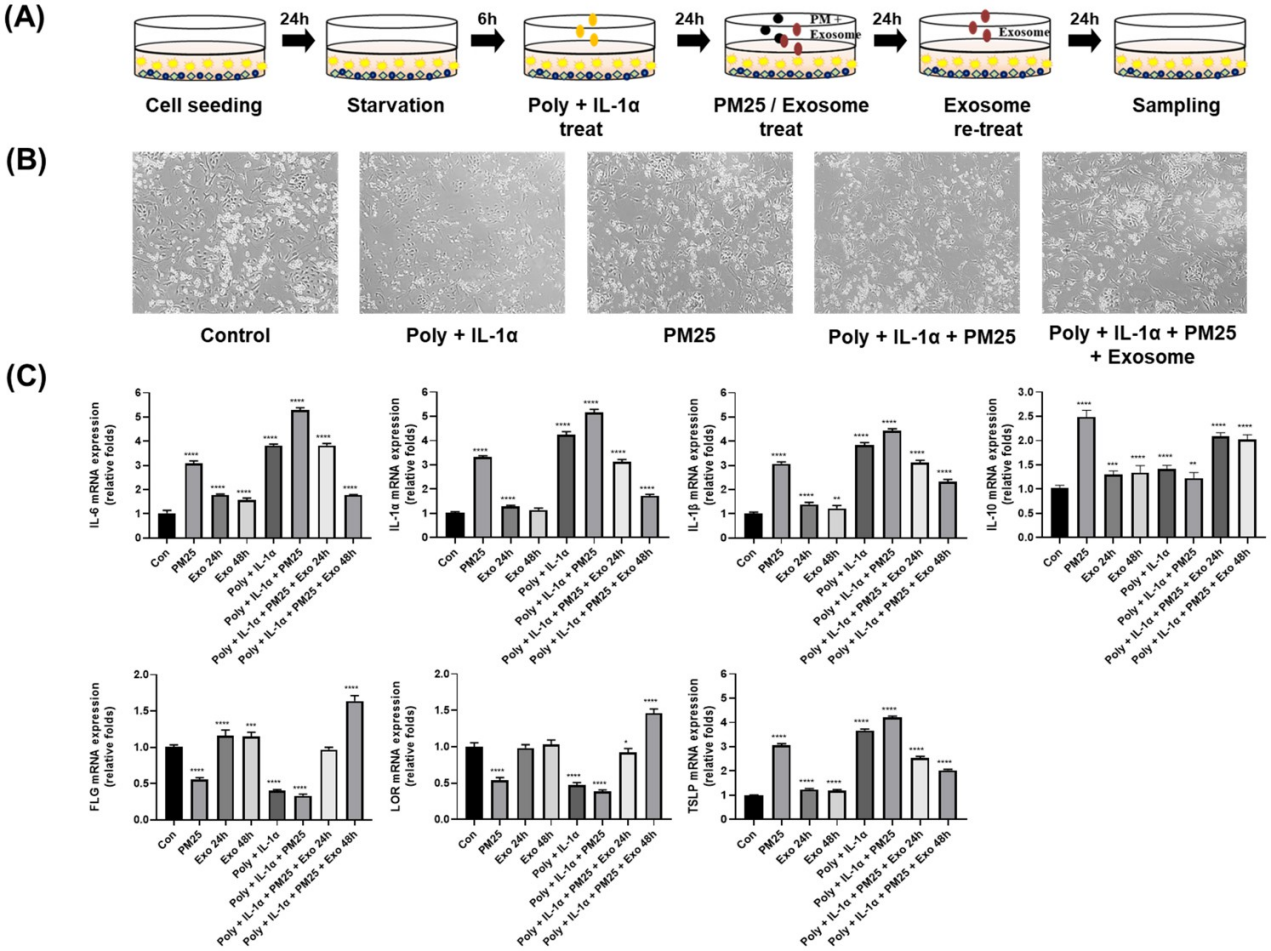
Effect of ASC-exosomes on the mRNA expression level of pro-inflammatory cytokines and skin barrier genes in the AD-like triple-cell model. (A) Schematic representation of the PM and ASC-exosome processing in an AD-like triple-cell model. (B) Microscopic examination of cellular morphologies after treating with Poly I:C, PM, and ASC-exosomes. (C) The mRNA expression levels of IL‑6, IL‑1α, IL‑1β, IL‑10, LOR, FLG, and TSLP are presented as relative fold change compared with un-treated cells. The data are shown as the mean ± standard deviation for triplicate experiments. **** *p* < 0.0001, *** *p* < 0.001, ** *p* < 0.01, *p < 0.05, compared with control.

An AD-like triple-cell model was established through Poly I:C and IL-1α treatment of triple-cells. Cells were pretreated with Poly I:C and IL-1α for 24 hours, followed by treatment with ASC-exosomes and PM (25 μg/cm^2^) for 24 and 48 hours (Fig 2A and 2B). qPCR was used to analyze the mRNA expression levels of IL-10, IL-6, IL-1β, IL-1α, LOR, and FLG, and the results were normalized to GAPDH. It was found that PM increased the mRNA expression of pro-inflammatory cytokines IL-6, IL-1β, and IL-1α and decreased the anti-inflammatory cytokine IL-10. PM also suppressed the expression of skin barrier genes FLG and LOR. However, when the AD-like triple-cell culture system was treated with ASC-exosomes, the pro-inflammatory cytokines decreased, and the skin barrier genes increased (Fig 2C). These results show that ASC-exosomes reduced the mRNA expression of pro-inflammatory cytokines and rescued the effects on skin barrier genes.

### Effects of ASC-exosomes on the protein levels of pro-inflammatory cytokines and a skin barrier protein in an AD-like triple-cell model

**Fig 3:**
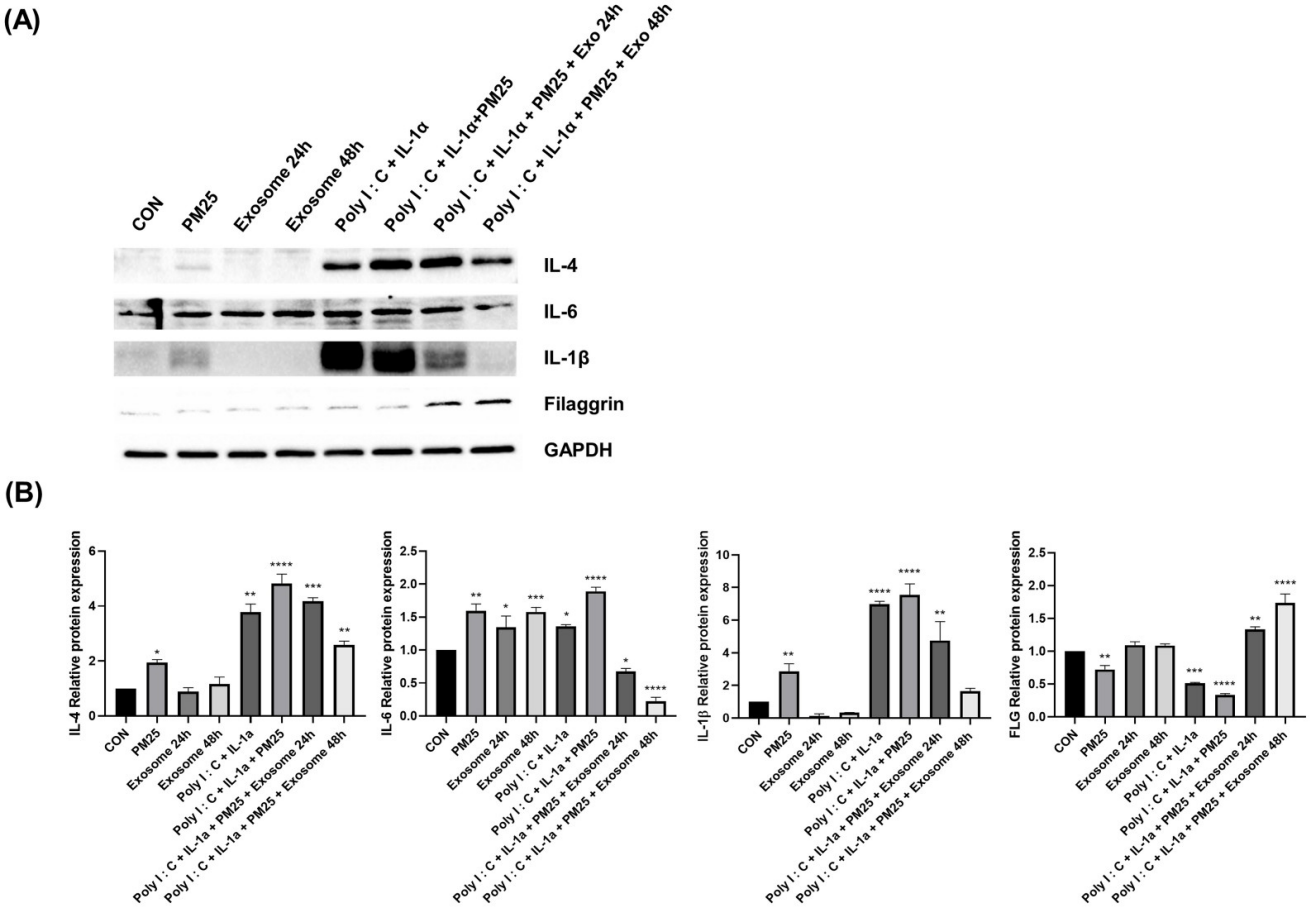
Effect of ASC-exosomes on the protein expression of pro-inflammatory cytokines and a skin barrier protein in a PM-treated AD-like triple-cell model. (A) The protein expression levels of IL-4, IL-6, IL‑1β, and FLG were determined using western blotting. (B) Quantitative results of the western blot analysis. The data are shown as the mean ± standard deviation for three independent experiments. **** *p* < 0.0001, *** *p* < 0.001, ** *p* < 0.01, * *p* < 0.05, respectively.

Western blotting was confirmed to evaluate the effect of PM and ASC-exosomes on the protein expression of pro-inflammatory cytokines and a skin barrier protein. The triple-cell culture system was pretreated with Poly I:C and IL-1α for 24 hours, followed by treatment with PM and ASC-exosomes for 24 hours. After that, the cells were further treated with exosomes for 24 hours. We found that PM increased the protein expression of the pro-inflammatory cytokines IL-4, IL-6, and IL-1β and decreased the expression of the skin barrier protein FLG (Fig 3A and 3B). In contrast, ASC-exosome treatment reduced the protein expression of the IL-4, IL-6, and IL-1β and increased the expression of FLG (Fig 3A and 3B).

### Effects of ASC-exosomes on PM-induced inflammation and skin barrier markers in an AD-like triple-cell model via immunofluorescence staining

**Fig 4:**
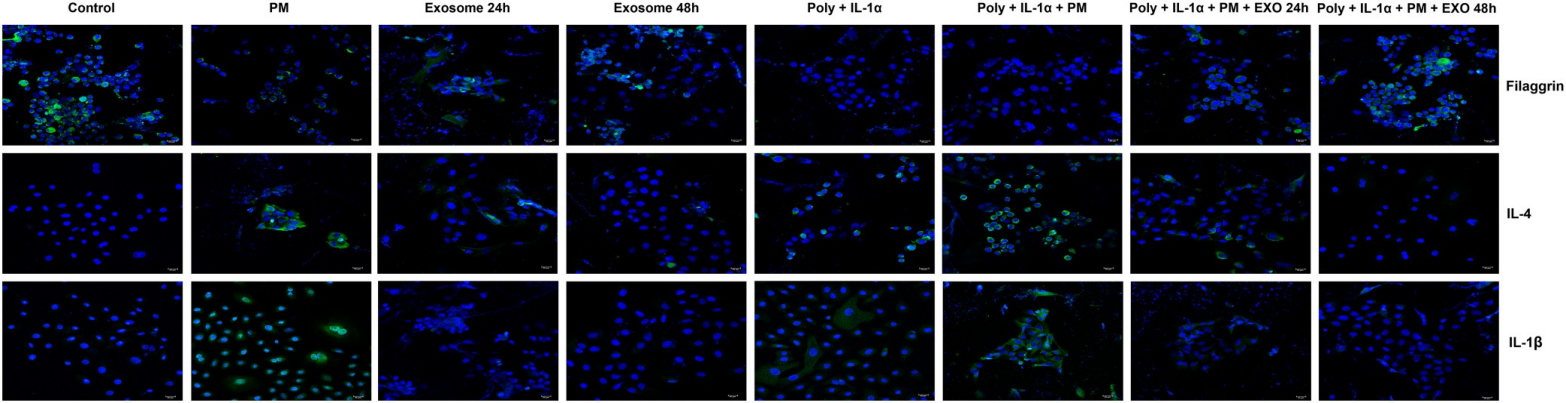
The immunofluorescent staining of pro-inflammatory cytokines and a skin barrier protein in the PM and ASC-treated AD-like triple-cell model. Green fluorescence indicates the expression of IL-4, IL-1β, and FLG in response to Poly I:C, IL-1α, PM, and ASC-exosomes. Nuclei are labeled with 4,6-diamidino-2-phenylindole (DAPI, blue). Scale bar denotes 20 μm length.

The expression of IL-4, IL-1β, and FLG in our AD-like triple-cell model was assayed using immunofluorescence staining analyses. The fluorescent staining of IL-4 and IL-1β was increased in the triple-cell culture system treated with PM (Fig 4). These cells also displayed a decrease in the skin barrier protein FLG (Fig 4). However, when these cells were treated with ASC-exosomes, the fluorescent staining of the pro-inflammatory cytokines and the skin barrier protein recovered (Fig 4).

## Discussion

In this study, we aimed to investigate the therapeutic effects of ASC-exosomes on PM-induced inflammation and skin barrier damage using an AD-like in vitro cell model. ASC-exosomes elicit multiple effects because they contain many substances related to stem cell differentiation, regeneration, and healing [33]. Exosomes have been shown to relieve AD symptoms, regenerate the skin barrier, and decrease tissue infiltration of mast cells [34]. In our previous study, we developed a new in vitro AD-like triple-cell culture model using human keratinocytes, dermal fibroblasts, and mast cells [12]. As is well known, keratinocytes and dermal fibroblasts are the main cells that comprise the epidermis and dermis of the skin, and the crosstalk between two cells plays a crucial role in skin homeostasis, cell differentiation, and inflammatory responses [35]. In particular, keratinocytes play an important role in atopic inflammation by secreting various proinflammatory mediators and chemokines. In addition, mast cells are one of the major effector cells of allergic responses and are suggested to be involved in atopic inflammation by detecting the surrounding environment. Furthermore, this model provides high degree of cell-to-cell contact, mimicking cellular interactions within the atopic skin [36]. To better examine the potential of exosomes as a therapeutic agent in PM-exacerbated AD, we treated our AD-like cell model with PM to exacerbate inflammation and reduce barrier markers. PM exposure has been known to increase inflammation and the emergence of skin diseases, including AD and psoriasis [15, 29]. In previous studies, PM-induced effects were evaluated using AD-like models consisting of single-cell cultures or triple-cell cultures containing keratinocytes, fibroblasts, and mast cells [12, 29, 31]. In the triple-cell culture model, increased expression of pro-inflammatory cytokines (IL-6, IL-1α, IL-1β, and TNF-α) and decreased expression of skin barrier proteins, including filaggrin and loricrin, was observed after exposure to PM [12]. The cytokine secretion pattern was consistent with the inflammatory responses by PM previously identified in AD skin, suggesting that the triple-cell culture model is a reliable model that visualizes the cell interactions associated with AD [5, 8]. In the present study, the increase of these inflammatory factors was attenuated, and levels of the skin barrier marker, FLG, were recovered in cultures treated with ASC-exosomes. Our results suggest that ASC-exosomes can be effective for improving inflammation in PM-exacerbated AD and restoring the skin barrier function [7, 37].

The triple-cell culture AD-like cell model could increase our understanding of the underlying cellular mechanisms of AD and provide evidence about the efficacy of treatments. The effects of ASC-exosomes in inhibiting inflammatory responses and restoring a skin barrier marker seen in this study are consistent with effects seen in previous animal studies [18, 28, 38], suggesting that the AD-like cell model can contribute to clinical research evaluating the therapeutic utility of exosomes for various skin diseases in the future. However, the exact molecular signaling pathways by which ASC-exosomes exert these anti-inflammatory effects and improve skin barrier function were not elucidated, which is one of the limitations of our study. Furthermore, well-designed animal and human studies are needed to confirm the efficacy of ASC-exosomes in improving AD. There may be concerns about the results of this study because the exosomes used in this study were derived from a single donor. However, the levels of 37 surface-labelled proteins in the 11 different batches of ASC-exosomes used in this study showed a high degree of consistency [21]. The results suggest that the TFF-based ExoSCRT^™^ technology facilitated the reproducible production of ASC-exosomes of a stable size and identity with batch-to-batch consistency. Therefore, we believe that the results of this study can be interpreted as effects of ASC-derived exosomes.

Recently, many novel bio-drugs targeting AD (such as dupilumab and JAK-inhibitors) have been developed with improved efficacy and fewer side effects than conventional systemic immunosuppressants [39]. However, these drugs are expensive, and the improvement rate is around 50–60%, making fundamental treatment still impossible. ASC-exosomes are expected to present a new paradigm for treating the fundamental causes of AD as a single drug or through combination therapy with existing treatments. ASC-exosomes may, therefore, improve the efficacy of existing therapies against inflammation, skin barrier damage, and itching.

In conclusion, our results confirm that PM-exacerbated inflammation and skin barrier damage were alleviated by ASC-exosomes using an AD-like triple-cell model. These findings will aid in discovering potential therapeutic targets using ASC-exosomes in AD; however, further research is needed to elucidate the precise mechanisms of ASC-exosome treatment in AD and to investigate the in vivo effects using animal models and clinical studies.

## Supporting information

S1 Fig Characterization of ASC-exosomes. Representative histogram of particle concentration and size distribution of ASC-exosomes measured by nanoparticle tracking analysis.
(TIF)

S2 Fig Representative Cryo-TEM image of ASC-exosomes. Scale bar: 200 nm.
(TIF)

S3 Fig Surface signature markers of ASC-exosomes quantified by flow cytometry. (TIF)
