# Supplementary figures and images for: Adipose tissue-derived exosomes alleviate particulate matter-induced inflammatory response and skin barrier damage in atopic dermatitis-like triple-cell model
Source: PLoS One. 2024 Jan 19;19(1):e0292050. doi: 10.1371/journal.pone.0292050 (PMC10798485; doi:10.1371/journal.pone.0292050)

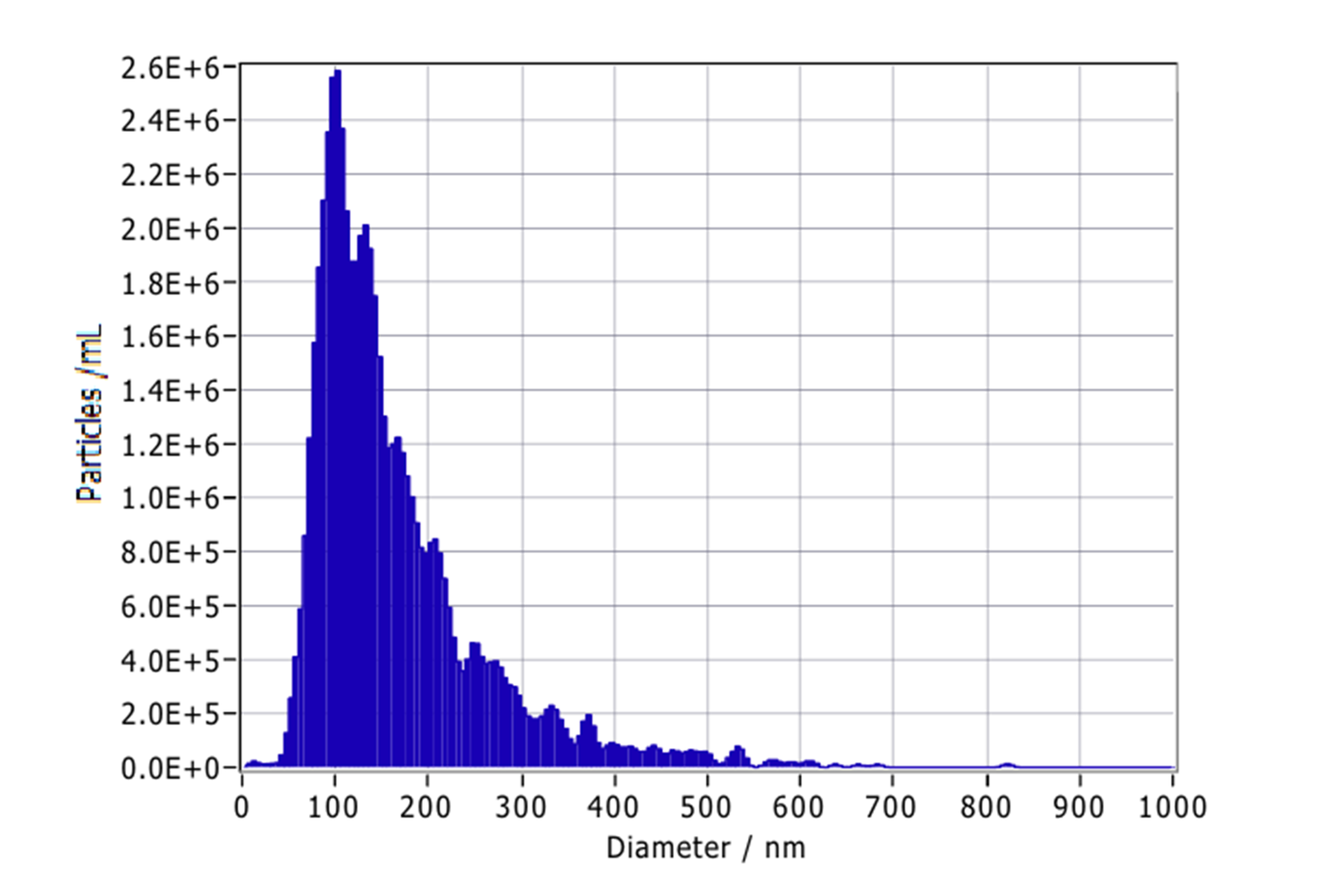

Supplement: S1 Fig — Representative histogram of particle concentration and size distribution of ASC-exosomes measured by nanoparticle tracking analysis. (TIF) [file pone.0292050.s001.tif]

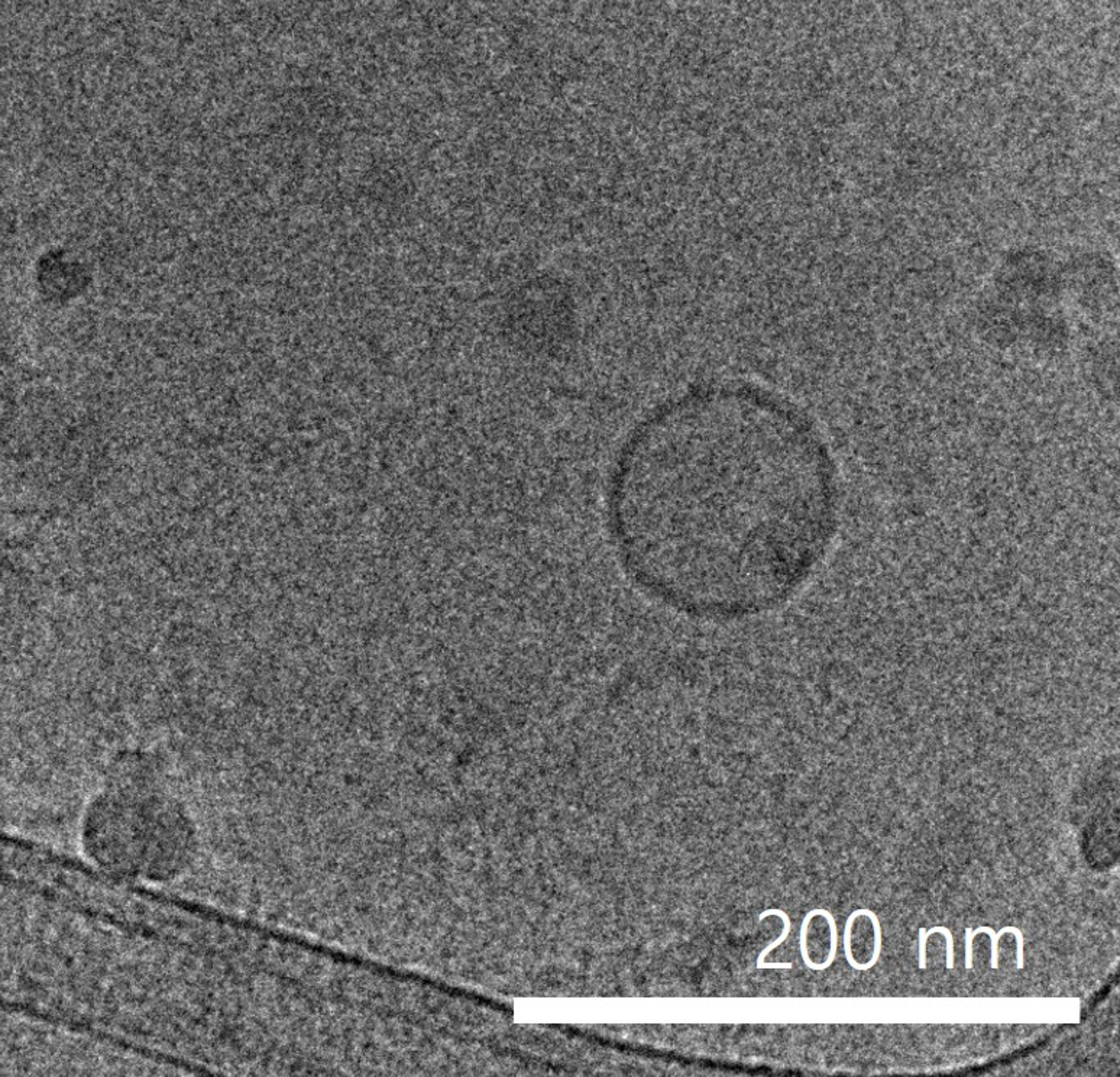

Supplement: S2 Fig — Scale bar: 200 nm. (TIF) [file pone.0292050.s002.tif]

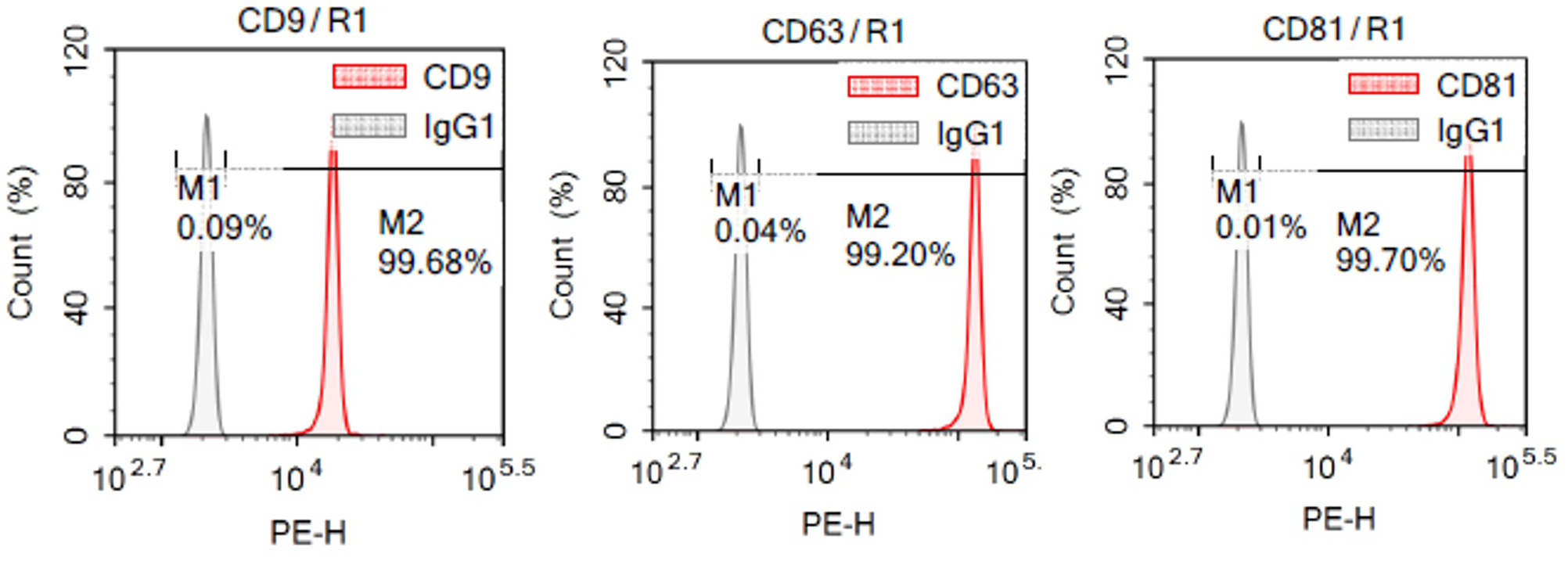

Supplement: S3 Fig — (TIF) [file pone.0292050.s003.tif]
